# Man, You Might Look Like a Woman—If a Child Is Next to You

**DOI:** 10.5709/acp-0174-y

**Published:** 2015-09-30

**Authors:** Aenne A. Brielmann, Justin Gaetano, Margarita Stolarova

**Affiliations:** 1 Department of Psychology and Zukunftskolleg, University of Konstanz, Germany; 2 Cognitive Neuroscience Research Cluster, School of Health and Human Sciences, Southern Cross University, Coffs Harbour, Australia

**Keywords:** male-bias, gender categorization, social perception, sex, social context

## Abstract

Gender categorization seems prone to a pervasive bias: Persons about whom null or ambiguous gender information is available are more often considered male than female. Our study assessed whether such a male-bias is present in non-binary choice tasks and whether it can be altered by social contextual information. Participants were asked to report their perception of an adult figure’s gender in three context conditions: (1) alone, (2) passively besides a child, or (3) actively helping a child (*n* = 10 pictures each). The response options *male*, *female* and *I don’t know* were provided. As a result, participants attributed male gender to most figures and rarely used the *I don’t know* option in all conditions, but were more likely to attribute female gender to the same adult figure if it was shown with a child. If such social contextual information was provided in the first rather than the second block of the experiment, subsequent female gender attributions increased for adult figures shown alone. Additionally, female gender attributions for actively helping relative to passive adults were made more often. Thus, we provide strong evidence that gender categorization can be altered by social context even if the subject of gender categorization remains identical.

## Introduction

No other social category is used as early, automatically, and pervasively as gender (Stangor, Lynch, Duan, & Glas, 1992; Weisman, Johnson, & Shutts, 2014). Gender attributions can have immense consequences, as gender stereotypes are still present (Seem & Clark, 2006) and contribute to the prevailing inequality between men and women (Brandt, 2011; Gaucher, Friesen, & Kay, 2011). Adding momentum to this notion, people readily attribute a gender to a person even in the absence of explicit gender cues. First described more than 30 years ago (Silveira, 1980) as a *people* = *male-bias* (in short: male-bias), early investigations of this effect were conducted using written descriptions of persons (e.g., Hamilton, 1991; Merritt & Kok, 1995). The effect has since appeared in many studies of human visual processing, suggesting that the underlying mechanisms are not necessarily linked to the generic use of male pronouns common in many languages.

In studies of visual gender categorization, male-bias has often been considered a nuisance phenomenon in need of circumvention by using bias-free statistics, or by feminizing stimuli to create a truly gender-ambiguous condition (Brooks et al., 2008; Hacker, Brooks, & van der Zwan, 2013; Troje, Sadr, Geyer, & Nakayama, 2006; Troje & Szabo, 2006). A robust male-bias can also emerge as a side-effect when participants categorize faces (Armann & Bülthoff, 2012; Johnson, Freeman, & Pauker, 2012; Wild et al., 2000), and may be enhanced by the emotionality of the faces (Bayet et al., 2015; Hess, Adams, Grammer, & Kleck, 2009). Independent of cues like emotion or pigmentation, ambiguous face (Davidenko, 2007), body (Johnson, Iida, & Tassinary, 2012) and hand (Gaetano, van der Zwan, Blair, & Brooks, 2014) shapes, as well as point-like walkers (Schouten, Troje, Brooks, van der Zwan, & Verfaillie, 2010) also have produced the male-bias.

To our knowledge, one study contradicts the general existence of a male-bias in gender categorization: Johnston, Miles, and Macrae (2008) studied how women’s gender categorization could vary as a function of ovulation and found that participants were more likely to misjudge male faces as female than make the opposite error, independent of ovulation stage. This finding is unlikely to stem from the exclusively female participants, as other studies found no difference in the male-bias of men and women (e.g., Schouten et al., 2010; Wild et al., 2000). Interestingly, Johnston and colleagues used the same argument to explain this female-bias as had been used to explain male-bias before by Wild and colleagues: That is, misidentifying either sex as the other might be associated with higher social costs, such as missing the opportunity for finding a mating partner. Thus, because this line of argument can be used to account for male- or female-bias, it seems likely that it is not a definitive explanation for either.

A different justification for the existence of male-bias is that male gender attribution serves as a default response because female gender is identified by means of the absence of male gender cues (Hess et al., 2009). Yet again, the reverse argument, namely that male-bias occurs because male is defined as the absence of female cues, has also been made (Intons-Peterson, 1988). Moreover, both arguments seem to ignore the possibility that male-bias could result from differences in variances of physical gender cues and not their mere presence or absence: If the spread of the distribution was broader for male than for female gender cues, this could lead to a higher probability of perceiving an ambiguous cue as male (cf. Clifford, Mareschal, Otsuka, & Watson, in press). So far, however, this alternative explanation has not been empirically tested. In summary then, it seems that male-bias is prevalent, yet has so far been ill-described, insufficiently understood, and explained. The current study addresses this divide, which might also help to overcome the challenges male-bias poses to the creation of standardized stimulus material (Todorov, Dotsch, Porter, Oosterhof, & Falvello, 2013).

One prominent gap in the explanation of the male-bias is that previous research has not answered the question whether it results from mere default responding (as argued by Wild et al., 2000) or from a biased default percept (Hess et al., 2009; Intons-Peterson, 1988). One reason for this shortcoming is that participants are generally only provided bipolar choice options: *male* versus *female*. In one study (Gaetano et al., 2014), participants were asked to indicate yes or no whether a certain stimulus was male, and separately whether a stimulus was female, instead of the classical male-female categorization task. As a consequence, participants were more likely to assign male gender when targeting male hands, as well as less likely to assign female gender when searching for female hands. Whereas such a tendency to identify male hands can be expected of participants who prefer the label *male* over *female*, such a preference is uninformative with respects to the yes or no responses to female targets. Male-bias then—at least in response to silhouette hand shapes—appears to involve more than a preference for assigning *male* versus *female* labels. Nevertheless, simple binary key press responses force participants to arbitrarily opt for a male favoring response when uncertain and cannot measure participants’ confidence. One method that allows more elaborate analyses of uncertainty would be to measure the trajectory of reaching movements towards the two response options (Quek & Finkbeiner, 2014), albeit this method does not give participants the possibility to decide for an intermediate judgment. Another way for taking uncertainty into consideration—one that is easier to implement than measurement of movement trajectories—would be to provide a third *neutral* response option reflecting uncertainty. Even though an option alongside *male* and *female* (or *yes* and *no*) may not guarantee its selection when the participant is uncertain, and this limitation is unpacked further in the Discussion, the assertion that male-bias is an artifact of the choice between only a male- and female-response becomes explicitly testable with the addition of a third option. The present study aimed to broaden our understanding of gender categorization and male-bias by allowing participants to use three response options: *male*, *female* and **I don’t know**.

The fact that male-bias appears in a variety of perceptual tasks implies that gender categorization is a multi-modal process. Considering the privileged and fundamental role of gender in human interaction (Stangor et al., 1992), it seems likely that gender categorization is governed by perceptual as well as cognitive processes. Earlier research has shown that written person descriptions set in a business context promote a higher male-bias relative to educational or interpersonal contexts (Merritt & Kok, 1995), and that mothers’ male-bias in choosing pronouns for child-like animals in picture books decreased if characters were shown in a social context with an adult (De Loache, Cassidy, & Carpenter, 1987). More recent research has mainly concentrated on gender categorization of a narrower set of stimuli—that is, of faces. Systematic influences on face gender categorization so far include emotional expressions (Bayet et al., 2015; Becker, Kenrick, Neuberg, Blackwell, & Smith, 2007; Hess et al., 2009), face race (Johnson, Freeman, et al., 2012), additional information in form of a male or female name (Huart, Corneille, & Becquart, 2005), and even proprioceptive toughness experienced by participants (Slepian, Weisbuch, Rule, & Ambady, 2011). In addition to these stimulus-related aspects, social desirability (or social approval) may also affect gender categorization and contribute to context effects. Social desirability or social approval effects stem from a tendency of participants “to portray themselves in keeping with perceived cultural norms” or “the need to obtain a positive response in a testing situation” (Adams et al., 2005, p.389). Within-subject comparisons in a gender categorization task might therefore reflect participants’ inclination to respond to different conditions in a way they believe to be appropriate rather than their actual perceptions of gender.

Taking into account these potential effects of context information and social desirability, gender categorization can be conceptualized as a dynamic integrative process to which not only multiple levels of perception, but also higher levels of cognition and stereotypes contribute (Freeman & Ambady, 2011; Johnson, Lick, & Carpinella, 2015; Johnston et al., 2008). It is all the more surprising then that current research almost exclusively focuses on gender categorizations of individual faces and has not re-examined earlier effects of gender stereotypical contexts reported two and more decades ago (De Loache et al., 1987; Merritt & Kok, 1995). To that end, our study investigated the extent by which perceptions of gender from pictures of adult figures are altered by context information—specifically the presence or absence of a child, as well as the active involvement of the adult in helping a child. Social desirability was explicitly considered as one factor potentially contributing to the presence or strength of a male-bias.

Hence, the goal of the present study was to determine whether: 1) the male-bias will still arise for drawings of human figures devoid of specific gender cues, given that a third response option **I don’t know** is provided, and 2) social context information (i.e., the presence of a child accompanying a target figure) can alter gender perception of visual stimuli. Our study used stimuli controlled regarding all other content and lower-level stimulus properties, while varying the social context systematically. Participants were asked for their subjective gender attributions regarding adult figures shown in three different context conditions: alone, passively present next to a child, or actively helping a child. We expected that participants would show a male-bias at least for pictures of adults alone. Moreover, given that the presence of a child is a feminine-stereotyped context, we hypothesized that male-bias would be reduced when adults were depicted with child. Last, we also expected that seeing the adult actively helping the child in a nurturing rather than dramatic context would further decrease the male-bias, similar to the educational context in Merritt and Kok’s (1995) study and particularly because gender imbalances in care-giving remain large even today (e.g., Barone, 2011). A smaller control experiment served to take potential effects of presentation order and social desirability into consideration.

## Experiment 1

### Methods

#### Ethics approval

This study was given formal ethics approval by the Ethics Committee of the University of Konstanz (July 31, 2013) and by the Dean of the Faculty of Society and Economics, Rhine-Waal University of Applied Science (October 1, 2013). All participants signed written informed consent according to the Declaration of Helsinki.

#### Stimuli

**Figure 1.**
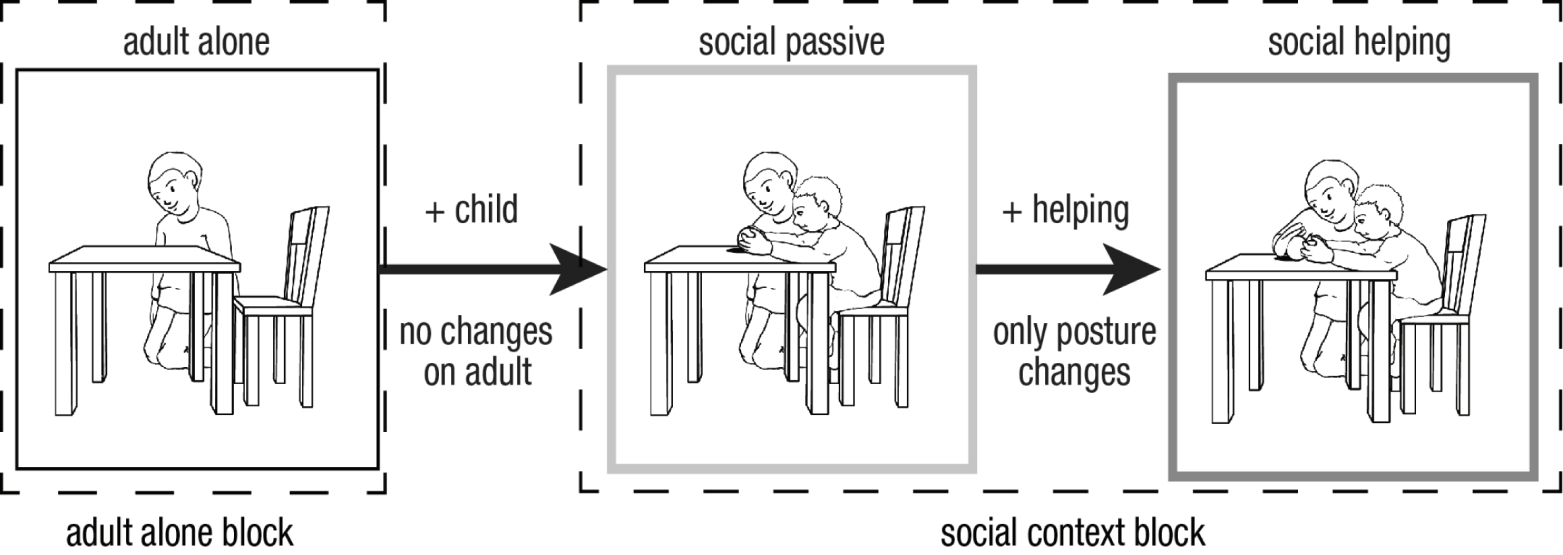
Stimuli for the three different conditions for one example situation. Pictures were generated in order to ensure maximum similarity between conditions. Arrows’ labels describe changes made for generating pictures with differing social context. Dashed frames group context conditions according to blocks within which pictures were randomized.

An extension of the NeoHelp Stimulus Set (Brielmann & Stolarova, 2014a) was employed. All stimuli were black-and-white comic drawings of adults in everyday situations (800 × 800 px). The adult figures were drawn without explicit male or female gender cues: Each had a short haircut, average non-curvy figure, and wore wide pants and t-shirt. A total of ten different situations were shown (e.g., an adult kneeling next to a table and chair). Three variations of each situation were derived, *adult alone*, *social passive*, and *social helping*, resulting in a total of 30 stimuli. The *adult alone* condition provided no social context information and served as a baseline measure. In the *social passive condition*, the adult figure was shown next to a child who acted without assistance—for example, grabbing a ball on a table. In the *social helping condition* the adult was depicted actively helping the child to reach a goal—for example, pushing a faraway ball towards the child. Slight body posture changes were necessary to convey the differences between social passive and social helping conditions, otherwise the adult figures were identical across all conditions. Figure 1 shows pictures for all three conditions for one example situation. The complete stimulus set is available at https://osf.io/ijk8w.

#### Procedure and design

**Figure 2.**
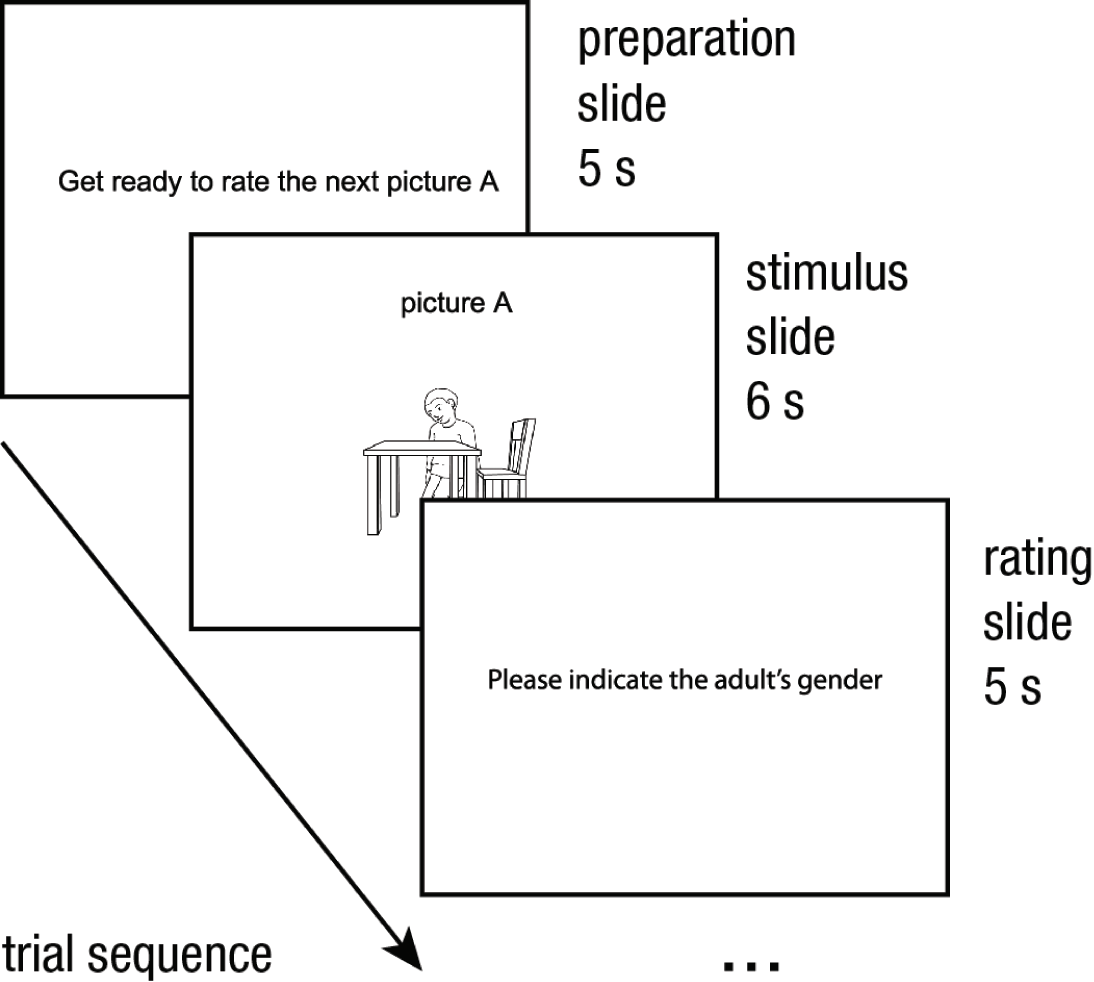
Time sequence for one example trial. All pictures were shown for 6 s, preceded by a 5 s preparation interval and followed by 5 s for responding. The order of pictures was pre-randomized within each block.

The experiments reported here were the first out of two which participants took part in during the time course of a regular university lecture. Each participant received one booklet and a separate consent form. The study was conducted in the language of the participants’ degree course (either English or German) at a German higher education institution. Stimuli were presented on projection screens in lecture halls. Each picture was shown for 6 s, preceded by a preparation slide shown for 5 s and followed by a slide prompting participants to indicate their decision in the provided booklets for 5 s (see Figure 2). Participants were free to change their answers, even though no specific instructions regarding changes were given and only unambiguously indicated final answers were included in the data. The order of picture presentation was quasi-randomized within two blocks, and was identical for all participants: one block contained the adult alone pictures (*n* = 10), the other one contained the social passive and social helping pictures (*n* = 20), intermingled such that pictures of the same situation were separated by at least one other picture. In the main study (Experiment 1), pictures of adults alone were first shown to participants (*n* =10), followed by pictures of child-accompanied adults (*n* = 20; 10 passive, 10 helping). We deliberately let participants rate the adult alone pictures first to collect baseline measures of gender attribution to a single figure without explicit gender cues. The exact order of stimuli is listed in the .text file available at https://osf.io/ijk8w. In a small control experiment (Experiment 2) the order of the two blocks was reversed, to test for effects of presentation order. Moreover, this control experiment also served as a partial control for effects of social desirability and social approval that cannot be ruled out by means of within-subject comparisons in Experiment 1. If male-bias in social context conditions would be on the level of or even lower for participants in Experiment 2 than for participants in Experiment 1, social context must affect male-bias over and above any possible—albeit not explicitly measured—effects of social desirability.

Prior to all analyses high frequency components were removed from the time series by applying a 15 Hz low pass Butterworth filter. CoP excursions were analyzed using two broad classes of parameters, related to (1) the amount of sway, and (2) the frequency contents of sway.

#### Participants

A total of 276 undergraduate students took part in Experiment 1; the complete raw data is available at https://osf.io/m5ciw/. Only participants with normal or corrected to normal vision were included in our analyses, leading to the exclusion of 36 participants (missing information about vision impairments: *N* = 26; uncorrected vision impairments: *N* = 10). Another 12 participants were excluded because answers were missing for more than three pictures in one or more conditions. Thus, data of 228 participants (*M*_age_ = 21.5 years, *SD* = 3.6, 25.4% men) were analyzed. The left column of Table 1 lists the complete population characteristics. Language of the booklet was not included in the main analyses (but can be found in the Supplementary Material) as the number of participants receiving German and English material was nearly equal and no hypotheses regarding the effects of language were made. Gender of the participants was included in preliminary analyses, but had no systematic impact on any aspect of the response behavior (see Supplementary Material for details).

**Table 1.** Population Characteristics

		Experiment 1	Experiment 2	Comparison sample
*N* total		228	21	21
% male		25,44	14,29	14,29
Handedness	Right	193	20	20
	Left	20	1	1
	Both	7	0	0
	Missing	8	0	0
% German citizens		80,26	90,48	95,24
Native language(s)	German	189	21	21
	English	17	0	0
	Other/missing	22	0	0
Field of study	Alternative tourism	30	0	0
	Education	62	19	19
	International business	50	2	2
	International relations	74	0	0
	Other	9	0	0
	% German study language	47,81	90,48	90,48

*Note*. It was possible to indicate more than one native language

#### Data analyses

All analyses were conducted using R (version 3.0.2); the analysis code can be accessed at https://osf.io/t3b5m/. For each participant, proportions of gender attributions were calculated as a function of stimulus condition (*adult alone*, *social passive*, *social helping*). As we were interested in the relative frequency of male compared to female gender attributions independent of the proportion of *I don’t know* responses, the difference between the proportion of *male* and *female* responses was calculated for each participant per condition. This difference served as an indicator of the strength of the male-bias.

Preliminary analyses were conducted to investigate picture sequence effects within each of the two blocks. To that end, Pearson correlations were computed between trial number and the difference between male and female gender attributions as well as the proportion of *I don’t know* responses.

As main analyses, we first calculated 95% between-subjects confidence intervals (CIs) around the mean difference in male-bias and proportion of I don’t know responses between picture categories. To illustrate our findings and substantiate the former results, we calculated 95% within-subject CIs around the gender attribution difference rates and around proportions of *I don’t know* responses per context condition. Within-subject CIs were calculated via the approach proposed by Cousineau (2005), with a correction by Morey (2008) and R-code provided by Baguley (2012). To compare data between experiments, simple between-subjects 95% CIs were calculated. The degree of overlap between CIs formed the basis of analysis, as this conservative approach is considered to yield more information-rich interpretations of data compared to the dichotomous assessment of *p*-values (e.g., Cumming, 2012, 2014). For independent groups, 95% CIs whose extremes just touch upon a meaningful difference even given a very conservative criterion (approx. *p* = .006), while the most common criterion for significance (*p* < .05) is approximated if 95% CIs for independent groups overlap by up to half of the (averaged) margin of error (Cumming & Finch, 2005). As a measure of effect size for differences between proportions, we calculated Cohen’s *d* with Hedge’s correction using the function *cohen.d* in the R-package *effsize*.

Second, the proportion of response alterations between context conditions for each situation was analyzed. Alterations were counted between adult alone and social passive as well as between social passive and social helping pictures within the same situation. These alterations were then sorted into four different categories: *no change*, *change to female*, *change to male*, and *change to I don’t know*. Response alterations that included missing values were excluded from analyses. Finally, 95% CIs of the proportion of alterations falling into each category were calculated.

### Results

#### Social context influences gender attributions but does not eliminate the male-bias

First, trial sequence within each block did not influence the difference between proportions of male and female responses (adult alone block: *r*(8) = .30, *p* = .40, 95% CI [-.41, .78]; social context block: *r*(18) = -.11, *p* = .64, CI [-.53, .35]) or proportions of *I don’t know* responses (adult alone block: *r*(8) = .05, *p* = .89, CI [-.60, .66]; social context block *r*(18) = .22, *p* = .36, CI [-.25, .60]). Also, data analyses for men and women separately yielded very similar results (see Supplementary Material for details) and, hence, we will report data for the complete sample.

**Figure 3.**
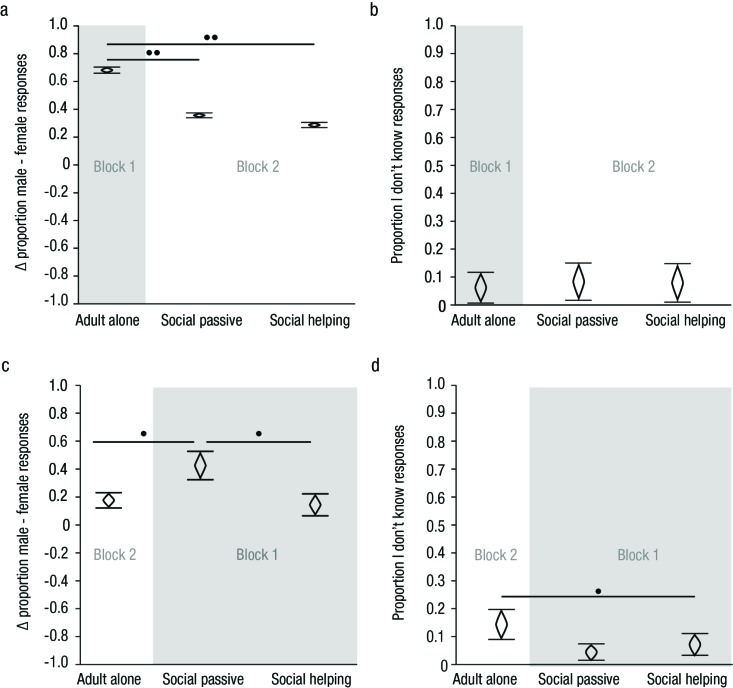
Mean difference between the proportion of male and female responses (a, c) and mean proportion of *I don’t know* responses (b, d) on the y axis for each context condition on the *x* axis in Experiment 1 (top) and the control Experiment 2 (bottom). Gray shading marks conditions that were shown in the first block of each experiment. Cat’s eyes represent 95% within-subject CI s. Non-overlapping CIs indicate meaningful differences between conditions. Dots mark magnitude of these differences; ••d >> 1.00, •d > 0.50.

Figure 3a displays the mean difference in proportions of male and female attributions with their within-subject CIs for each condition. As all of these CIs lay meaningfully above zero, a clear male-bias was evident in all three context conditions. As indicated by the CIs of differences not overlapping zero in the left half of Table 2, the presence of a child modulated the magnitude of difference between the amount of male and female responses: The likelihood with which male attributions were more frequent than female attributions was greater for pictures showing an adult alone compared to both social passive and social helping pictures by *d* = 1.07 and *d* = 1.25, respectively. Additionally, the male-bias was more strongly reduced in social helping compared to social passive context conditions, albeit to a lesser degree, *d* = 0.21.

**Table 2.** Mean Differences in Male-Bias and Proportions of *I Don’t Know* Responses Between Picture Categories and Their 95% Between-Subject Cls Within Each Experiment

	Experiment 1				Experiment 2			
	Male-bias		Proportion *I don’t* know		Male-bias		Proportion *I don’t* know	
Difference considered	MD	95% CI	MD	95% CI	MD	95% CI	MD	95% CI
Social passive—adult alone	**-.32**	[-.36, -.28]	**.02**	[.01, .04]	**.25**	[.08, .42]	**-.10**	[-.03, -.17]
Social helping—adult alone	**-.39**	[-.43, -.35]	.02	[.00, .03]	-.03	[-.14, .07]	-.08	[.00, -.15]
Social helping—social passive	**-.07**	[-.10, -.04]	.00	[-.02, .01]	**-.28**	[-.48, -.08]	.02	[-.02, .07]

*Note*. Male-bias refers to the difference between proportions of male and female responses, such that negative values here indicate a reduction of male-bias in the first relative to the second condition. Differences that lie meaningful above zero are highlighted in bold. MD = mean difference.

*I don’t know* responses were rare (6.2% to 8.4%) and occurred with similar frequency in social passive and social helping conditions (see Figure 3b and Table 2). The small difference between the proportions of *I don’t know* responses to adult alone and social passive pictures, mean difference (MD) = .02, *d* = 0.21, cannot account for the large difference between those conditions in the male-bias. Hence, changes in the difference between proportions of female and male responses cannot be attributed to changes in participants’ likelihood to choose the option *I don’t know*.

#### Change in responses across different context conditions

**Figure 4.**
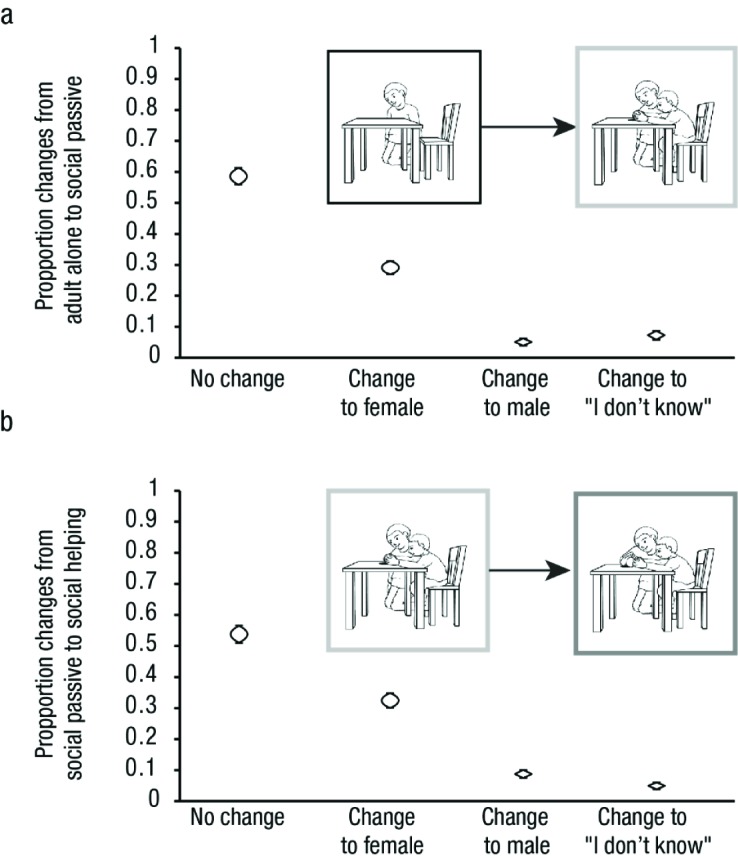
Proportion of response alterations within pictures of one situation in Experiment 1. Changes were counted and categorized between adult alone and social passive (a) as well as between social passive and social helping conditions (b). As in Figure 1, example pictures are framed according to condition (black = adult alone, light gray = social passive, dark gray = social helping). Cat’s eyes represent 95% CI s.

For each situation the adult figure was identical across all three context conditions, apart from slight, necessary changes in posture to convey the situational difference between social passive to social helping (see Figure 1). Thus, alterations of responses occurring above chance can be attributed solely to influences of contextual changes. Figure 4a illustrates the proportion of response alterations from adult alone to social passive, and Figure 4b from social passive to social helping pictures. Both patterns were highly similar. In absolute numbers, participants were most likely to remain constant in their gender attributions (> 50%). If a change in gender attribution for a given adult figure occurred from the adult alone to one of the two social conditions, the gender attribution most likely changed from *male* or *I don’t know* to *female* (29.1% and 32.5%; see Figure 4). Changes to *male* (5.0% and 8.8%) or *I don’t* know responses were much rarer (7.3% and 5.0%). As both of these alterations occurred with similar and very low frequency (see Figure 4), it is likely that they represent random rather than systematic response changes. These results illustrate that participants’ decreased male-bias for pictures showing the adult along with a child (see Figure 3a, b) truly emanates from participants switching their initial gender attributions for a given adult figure to female due to changes in social context.

## Experiment 2

In Experiment 1 we deliberately let participants rate the adult alone pictures first to enable collection of baseline measures of gender attribution to a single gender-ambiguous figure. In order to investigate the influence of the reversed order of presentation, the sequence of the stimuli blocks was altered for a small subset of participants (*N* = 21, see Table 1 for details). We herewith aimed to provide further evidence that social context was a major factor leading to systematic changes of gender attributions to female and were not primarily influenced by presentation sequence or social desirability alone.

### Methods

#### Ethics approval, stimuli, procedure and design

The procedures of Experiments 1 and 2 were identical in all respects but block sequence. Participants in Experiment 2 were first shown the 20 social context pictures and afterwards the ten adult alone pictures; picture sequence was identical and pre-randomized within blocks for both experiments. As in Experiment 1, trial sequence within one block did not influence male-bias (both |*r*| ≤ .29, both *p* ≥ .41) or proportions of *I don’t know* responses (both |*r*| ≤ .16, both *p* ≥ .50).

#### Participants

A total of 21 undergraduate students took part in Experiment 2. No participants had to be excluded from analyses according to the exclusion criteria also applying to Experiment 1. The right column of Table 1 lists the complete population characteristics.

### Results

#### Social context influences gender attributions independent of block sequence

A male-bias persisted throughout all three conditions (see Figure 3c) in Experiment 2 just as in Experiment 1. Meaningful differences in male-bias and proportions of *I don’t know* responses for this experiment are displayed in the right half of Table 2. In line with results from Experiment 1 participants in Experiment 2 exhibited a decreased male-bias for adult figures depicted as helping a child rather than being passively present next to him/her (see Figure 3c and Table 2), *d* = 0.85. Thus, social context alone influenced gender attributions in both experiments. However, participants were markedly less likely to assign male than female gender to adults in the adult alone compared to the social passive condition, *d* = -0.83, when they had seen the same figures in two kinds of social interaction with a child first. In fact, the difference between male and female gender attributions was comparable for social helping and adult alone pictures, *d* = 0.15. As opposed to Experiment 1, however, the reduction of the male-bias in adult alone compared to social passive pictures may have been partially due to a higher proportion of I don’t know responses for adult alone compared to social passive pictures (see Figure 3d and Table 2), *d* = 0.62. The analyses of response alterations within one situation below served to further clarify the relative contribution of female, male, and *I don’t know* responses to the difference between social passive and adult alone pictures.

#### Comparison of Experiments 1 and 2

In order to directly compare results of Experiments 1 and 2, we selected a comparison sample from Experiment 1 matched to participants of Experiment 2 with regard to gender, handedness, field of study, nationality, and study language (see rightmost part of Table 1). This comparison enables us to rule out that social context effects depend on block order. The corresponding 95% CIs used for comparison are shown in Table 3.

Male-biases for social context pictures were not meaningfully different for Experiments 1 and 2 as would be expected if sequence effects alone would cause an effect (see Table 3). Moreover, the male-bias was smaller for social helping pictures shown in the first block in Experiment 2 compared to the baseline measure for the male-bias as assessed in the first block of Experiment 1, *d* = -2.07. There was no such reliable difference between social passive pictures in Experiment 2 and adult alone pictures in Experiment 1, *d* = -0.73, possibly because participants in Experiment 2 contrasted social passive and social helping pictures more strongly with each other than participants in Experiment 1. However, the male-bias for adult alone pictures was much smaller if the social context block had been shown first, *d* = 2.34. Also, male-bias was moderately smaller in Experiment 2 compared to Experiment 1 for social helping pictures, *d* = 0.68, albeit this difference can be considered meaningful only when applying a less strict criterion than non-overlapping 95% CIs. Thus, changes in the male-bias for adult figures depicted alone in Experiment 2 followed a different pattern than in Experiment 1, but confirmed that social context information influences gender attributions.

**Table 3.** Means and Between-Subject 95% CIs For Male-Bias and Proportion of I Don’t Know Responses for All Picture Categories

	Male-bias				Proportion *I don’t know*			
	Experiment 1 Comparison sample		Experiment 2		Experiment 1 Comparison sample		Experiment 2	
Picture category	M	95% CI	M	95% CI	M	95% CI	M	95% CI
Adult alone	**.65**	[.55, .76]	**.18**	[.10, .26]	.09	[.04, .14]	.15	[.06, .24]
Social passive	.35	[.21, .48]	.43	[.25, .60]	.08	[.02, .14]	.05	[.00, .10]
Social helping	.34	[.20, .49]	.14	[.02, .26]	.06	[.02, .10]	.07	[.03, .12]

*Note*. Male-bias refers to the difference between proportions of *male* and *female* responses, such that higher values here indicate a stronger male-bias. Meaningful differences between experiments, as indicated by non-overlapping CIs, are highlighted in bold. Note that under application of the less strict difference criterion of overlap by up to half of the (averaged) margin of error, male-bias for social helping pictures is smaller in Experiment 2 compared to Experiment 1

#### Change in responses mirrors effects for proportions of responses

**Figure 5.**
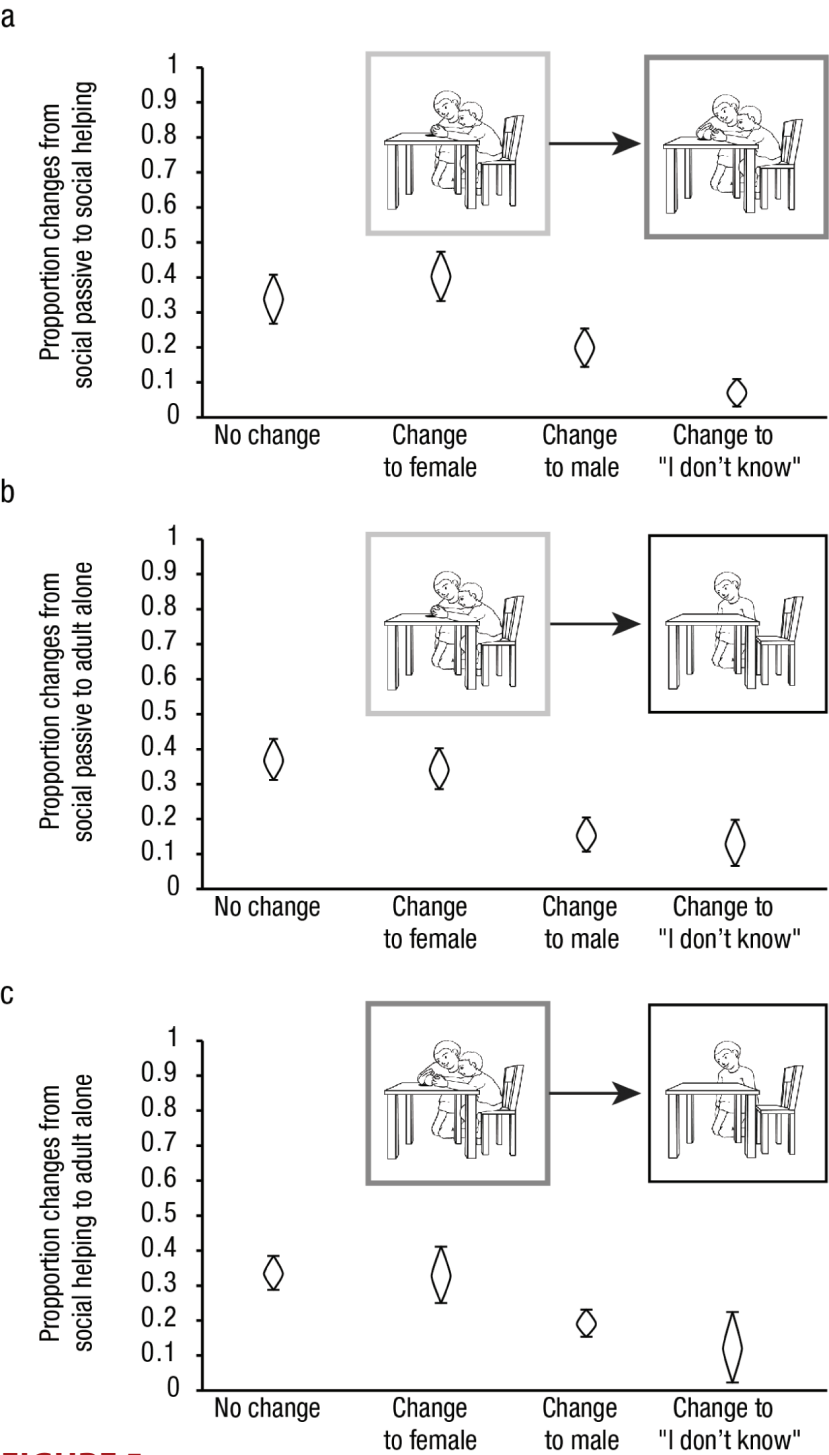
Proportion of response alterations within pictures of one situation in Experiment 2. Changes were counted and categorized between social passive and social helping (a), social passive and adult alone (b) as well as between social helping and adult alone conditions (c). As in Figure 1, example pictures are framed according to condition (black = adult alone, light gray = social passive, dark gray = social helping). Cat’s eyes represent 95% CI s.

As for Experiment 1, we verified that differences in proportional responses resulted from changes of responses to identical figures, by analyzing participants’ response alterations (see Figure 5). In contrast to Experiment 1, there was no absolute tendency of participants in Experiment 2 to most often remain constant in their gender attributions. When comparing gender attributions for social passive and social helping pictures we found stronger evidence that seeing an adult figure actively helping a child in a nurturing situation increases the likelihood that this figure is perceived as female (see Figure 5a). Given the higher proportion of female gender attributions to helping compared to passive adults, response alterations between social context and adult alone pictures fit the expected pattern given that previous gender attributions influence later ones: Participants switched to female rather than to male responses (see Figure 5b, c). These findings put forward the hypothesis that one attribution of female gender is sufficient for increasing the likelihood of female gender attributions in case that fewer gender cues are provided for subsequent gender attributions of an identical or very similar figure.

## Discussion

In a small control experiment (Experiment 2) seeing pictures with social context first led to a marked decrease in the male-bias for the same adult figures without social context presented afterwards. These results affirm findings from Experiment 1 in that the context in which a figure without explicit gender cues is shown can influence gender attributions. They expand the conclusions drawn from Experiment 1 by showing prior availability of social context information influences later gender attributions to the same figure shown without the context cues. Experiment 2 also confirmed that male-bias is even more reduced for pictures showing a helping adult figure compared to a passive one beside a child. Analyses of response alterations vindicate the assumption that the presence of a child leads to more female gender attributions compared to depictions of the same adult alone, even though overall male gender attributions prevail.

## General Discussion

The study presented here is the first to systematically investigate the male-bias using drawings of human figures that do not provide explicit gender cues. It makes three major contributions to the field of social visual perception. First, we show that a male-bias is evident for human adult figures depicted without explicit gender cues. Second, we provide evidence that this bias persists even when the alternative *I don’t know* is provided. Third, we demonstrate that the male-bias can be reduced—albeit not extinguished—by providing specific social context information. Whereas the first two findings provide novel and compelling evidence for a male-bias in visual perception, the third finding emphasizes the importance of information unrelated to the perceptual appearance of the to-be-categorized figure for gender categorization.

### Pictures of adult figures devoid of gender cues are predominantly perceived as male

The tendency to attribute male gender to gender-ambiguous stimuli has been reported in the methods section of many studies investigating gender categorization of visual stimuli (e.g., Davidenko, 2007; Hess et al., 2009; Johnson, Freeman, et al., 2012; Johnson, Iida, et al., 2012) while only sometimes being mentioned as a finding itself (Armann & Bülthoff, 2012; Gaetano et al., 2014; Schouten et al., 2010; Wild et al., 2000). Irrespective of its centrality within such studies, male-bias has been targeted exclusively via binary tasks that do not allow distinction of response biases from perceptual biases because a binary response format forces participants to opt for either male or female responses when uncertain. This study is the first to explicitly report male-bias in social visual perception while providing a response-alternative suitable to capture uncertainty: Participants attributed male more often than female gender to all of the adult figures across three different conditions and two sequences of presentation (see Figure 3a, c). *I don’t know* responses were infrequent and independent of category condition. The present experiments therewith showed that the male-bias for gender-ambiguous figures cannot be accounted for by dichotomous response heuristics alone (e.g., assigning male attributions to faces without clear female hairstyles, as argued by Wild et al., confirming a recent study that investigated gender categorization of silhouette hand shapes (Gaetano et al., 2014). In line with previous studies of gender categorization (e.g., Schouten et al., 2010; Wild et al., 2000), the gender of the participants themselves did not affect the presence or strength of the male-bias.

### Social context changes gender attributions to identical figures

The adult figures pictured in our stimuli were identical across social contexts, with only minor posture changes between social passive and social helping conditions. The stimuli depicted everyday situations and calculations of specific changes across conditions relied on comparisons within the variations of a situation across context conditions. We found that participants’ male-bias was strongly influenced by the context in which the figure was presented (see Figure 3a, c). In Experiment 1, we obtained baseline measures of male-bias by first presenting the adult figure without further context information; the participants were not aware that they would see the same adult figures across different context variations. The group average male-bias diminished when the adult figures were shown along with a child and most strongly so if the adult helped the child. These findings extend earlier demonstrations of gender stereotype consistent alterations in male-bias (Merritt & Kok, 1995; see also De Loache et al., 1987), and are consistent with the notion that gender stereotypes still prevail today (Seem & Clark, 2006). Findings from Experiment 2 ruled out a trivial explanation of these findings by means of presentation order, social desirability, or social approval alone (for a definition see, e.g., Adams et al., 2005, p.389). Such effects may have caused participants in Experiment 1 to, for example, (a) give more female ratings to the social context pictures because they thought they were expected to contrast between the adult alone block and the social context block, or (b) give more female responses towards the end of the experiment because they felt that they have to use this response option, too. Based on a very conservative criterion for differences between groups, participants’ male-bias was of similar strength in both experiments for both picture categories including a social context, no matter whether they were presented in the first or second block. Relaxing this criterion, one finds even more striking evidence for this claim: a moderately lower male-bias for social helping pictures in Experiment 2 compared to Experiment 1. Such a difference cannot stem from either social desirability or sequence effects. Also, a similar decrease in male-bias from social passive to social helping pictures was observed in both experiments. Both times pictures comprising these two social context categories were presented in random sequence and, therefore, the difference in male-bias between social passive and social helping pictures cannot stem from a sequence effect. Together with the results of the within-block correlation analyses conducted to assess per-trial effects of presentation sequence, these findings substantiate the assumption that the effects of social context cannot be attributed to order effects.

The comparison between Experiments 1 and 2 also revealed that male-bias was larger if the adult figures had been shown alone first, before participants were aware that there would be any social context. Phrased another way, male-bias was weaker if the sole adult figures were presented after the adult and child figure pairings. Hence, the impact of context on gender categorization was not only immediate, but persistent in that having once seen the same figure in a social situation elicited relatively more female gender attributions to the same figure presented alone later in the experiment. This effect, however, is not necessarily explained by stereotype effects alone. Rather, it also suggests another important contribution to gender attributions: Having made one female gender attribution may promote further female gender attributions to the same figure. Alternatively, the initial presentation of social contexts in general may lead to a reduction in male-bias for all subsequent adult figures presented alone. We cannot resolve this question on the basis of the present data, since we deliberately focussed analyses on changes in gender attribution towards identical adult figures, but it points to a useful question for further research: Does a specific social context, here the presence of a child, diminish male-bias only for the same representations of adults or does it extend to all comparable figures presented afterwards?

It may seem counter-intuitive at first glance that participants in Experiment 2 showed the strongest male-bias for social passive pictures and not for the adults depicted alone, as in Experiment 1. If one supposes that participants formed a reference frame for gender attributions after early presentations and without knowing that the same adult figures will be presented alone, it may have been the case that these participants perceived or felt the need to make a larger difference in their gender attributions for social passive and social helping pictures. The social passive condition was hence least affected, as participants likely created a reference frame from the first-shown social context block in Experiment 2. In other words, of the two social contexts, the social passive pictures would have appeared less female-stereotypical and so presumably are the ones to yield the higher male-bias. In summary, the specific social context always impacted gender categorization, but within the reference frame of information provided so far.

We were able to rule out the possibility that a decrease in male-bias was due to changes in the occurrence of *I don’t know* responses in two ways. First, systematic changes in *I don’t know* responses were either not observed or too small to account for the considerable changes in the strength of the male-bias. Second, analyses of response alterations unambiguously related the diminished male-bias to a meaningful increase in female gender attributions for stimuli showing identical adult figures. Moreover, response alterations in Experiment 2 strongly suggest that in the absence of objective gender cues, one prior female gender attribution to the same figure can augment the likelihood of subsequent female attributions (see Figure 5a, b).

In sum, our study shows that gender attributions to identical adult figures bearing no explicit gender cues can be altered in a stereotype-consistent way by providing social contextual information—here, the presence of a child or the act of nurturing or helping a child. It therewith extends and updates earlier findings that contextual information can alter perceived gender in a way consistent with stereotypes (De Loache et al., 1987; Merritt & Kok, 1995). This finding not only points out the influence of cognitive processes on gender categorization, it is also a demonstration of the pervasiveness of benevolent gender stereotypes in a well-educated, young population.

### Limitations and future directions

The highly controlled design of our stimuli can be regarded as the study’s greatest strength but also as a weakness. The black-and-white comic pictures are simpler and more abstract than naturalistic stimuli, hence, our findings should be generalized with caution. One advantage of these abstract, harmless stimuli is that they are well suited to study gender categorization in children, which could help elucidate the development of gender categorization. First results along this line indeed suggest that social context also modulates children’s gender attributions (Brielmann & Stolarova, 2014b) in line with the very recent finding of an angry-male-bias for faces in a population of children aged 5-6 years (Bayet et al., 2015). Also, our stimuli represent a class of real-life encounters with believed-to-be gender-ambiguous visual information rather well: child media. Given that adults’ use of gendered language may influence children’s development of sexist thoughts (Leaper & Bigler, 2004, but see also Friedman, Leaper, & Bigler, 2007, for contradictory findings), our results still have implications for everyday life. They directly point to a critical flaw in efforts to create gender-fair child media by providing protagonists that are devoid of gender cues.

Another restriction of our findings is that the context information provided only comprised children and the act of helping a child in nurturing, non-dramatic ways. It will be important to test whether other social contexts unrelated to children reduce the male-bias, or whether male rather than female gender attributions would be promoted by showing an act of helping that is physically taxing and might, hence, be more often seen in men (see Eagly, 1987, for a review on gender-differences in helping behavior). Related to this point, the stimuli we employed might be considered predominantly male or female, depending on whether that gender is defined by the absence of clear cues for the other (as argued by, e.g., Hess et al., 2009; Intons-Peterson, 1988). As our analyses focus on the changes in gender attribution between conditions, however, the above interpretations hold true, regardless of the default gender of the stimuli.

Finally, the possibility remains open that participants in our experiment hesitated to choose *I don’t know* as an answer, perhaps due to an expectation that an adult figure should be male or female. Considering the academic context of the study, participants may have considered the I don’t know option inappropriate or undesirable—despite instructions emphasizing that there were no right or wrong answers. Thus, this response option might not have strictly captured participants’ uncertainty as intended. Following studies from our lab include the label *no gender* as a response option to increase the likelihood that it is perceived as a viable response. Another option would be to directly measure participants’ response efficiency (a method, e.g., used by Quek & Finkbeiner, 2014) to estimate gender attributions’ certainty, or to use a rating scale from female to male, allowing participant to really rate the perceived masculinity and femininity on a continuous scale. Alternatively, the expectation—versus perception-based nature of the male-bias may further be probed by manipulating the ratio between male and female figures shown. If male-biased outcomes are the result of response tendencies and not of skewed perceptions, then participants’ bias scores should linearly track the ratio of female targets or male targets. For instance, participants inclined to respond male both (a) when in doubt and (b) irrespective of the relative frequency of male targets will appear most male-biased when targets are rare. In contrast, consider the outcome when male-bias were governed by perception: Measures would be smaller when targets are rare because the increased frequency of female lures presumably primes perception during ambiguous trials. As previous studies have adopted the unbiased ratio (e.g., Becker et al., 2007: Study 2; Bruce et al., 1993; Gaetano et al., 2014; Wild et al., 2000) that closely matches that found among real human populations (Central Intelligence Agency, 2014), manipulating this ratio in future studies will give a more precise answer to the question whether the male-bias can be accounted for by a response bias.

### Conclusion

This study is the first to report an in-depth investigation of a male-bias in gender categorization of complete human figures. A robust male-bias was observed even though a neutral *I don’t know* alternative was provided, rendering an explanation of the male-bias by means of a pure response bias unlikely. Despite the fact that drawings of adults were identical in all context conditions, the size of the male-bias decreased in two context variations including social interaction with a child. Participants were more likely to attribute female gender to adult figures shown along with a child, especially when the adult was actively helping a child. If such social context information was provided before the adult figure had been seen without a child, the higher likelihood of female gender attribution carried over to pictures providing no additional context information. Hence, we were able to show that gender categorization of visual stimuli that bear no explicit gender cues is influenced by contextual information in a gender stereotype confirming way, albeit not completely canceling a general male-bias.

## Author Note

This research was supported by the Zukunftskolleg of the University of Konstanz. We would like to thank all participants as well as the researchers assisting with data collection: Joana Chomakova, Marcus Kicia, and Jennifer Müller.
